# SLC29A1 single nucleotide polymorphisms as independent prognostic predictors for survival of patients with acute myeloid leukemia: an in vitro study

**DOI:** 10.1186/s13046-014-0090-9

**Published:** 2014-11-15

**Authors:** Haixia Wan, Jianyi Zhu, Fangyuan Chen, Fei Xiao, Honghui Huang, Xiaofeng Han, Lu Zhong, Hua Zhong, Lan Xu, Beiwen Ni, Jihua Zhong

**Affiliations:** Department of Hematology, Ren Ji Hospital, School of Medicine, Shanghai Jiao Tong University, 160 Pujian Road, Shanghai, 200127 China

**Keywords:** AML, SNP, Ara-C, Genotype

## Abstract

**Background:**

The mechanism behind poor survival of acute myeloid leukemia (AML) patients with 1-barabinofuranosylcytosine (Ara-C) based treatment remains unclear. This study aimed to assess the pharmacogenomic effects of Ara-C metabolic pathway in patients with AML.

**Methods:**

The genotypes of 19 single nucleotide polymorphisms (SNPs) of DCK, CDA and SLC29A1from 100 AML patients treated with Ara-C were examined. All the SNPs were screened with ligase detection reaction assay. The transcription analysis of genes was examined by quantitative real time polymerase chain reaction. The association between clinical outcome and gene variants was evaluated by Kaplan-Meier method.

**Results:**

Genotypes of rs9394992 and rs324148 for SLC29A1 in remission patients were significantly different from those in relapsed ones. Post-induction overall survival (OS) significantly decreased in patients with the CC genotype of rs324148 compared with CT and TT genotypes (hazard ratio [HR] = 2.997 [95% confidence interval (CI): 1.71-5.27]). As compared with CT and TT genotype, patients with the CC genotype of rs9394992 had longer survival time (HR = 0.25 [95% CI: 0.075-0.81]; HR = 0.43 [95% CI: 0.24-0.78]) and longer disease-free survival (DFS) (HR = 0.52 [95% CI: 0.29-0.93]; HR = 0.15 [95% CI: 0.05-0.47]) as well As compared with CT and TT genotype, patients with the CC genotype of rs324148 had shorter DFS (HR = 3.18 [95% CI: 1.76-5.76]). Additionally, patients with adverse karyotypes had shorter DFS (HR = 0.17 [95% CI: 0.05-0.54]) and OS (HR = 0.18 [95% CI: 0.05-0.68]).

**Conclusions:**

AML patients with low activity of SLC29A1 genotype have shorter DFS and OS in Ara-C based therapy. Genotypes of rs9394992 and rs324148 may be independent prognostic predictors for the survival of AML patients.

**Electronic supplementary material:**

The online version of this article (doi:10.1186/s13046-014-0090-9) contains supplementary material, which is available to authorized users.

## Background

Acute myeloid leukemia (AML), a heterogeneous disease with various clinical presentations, can be treated with 1-barabinofuranosylcytosine (Ara-C) or Ara-C combined with anthrocycline [1–3]. Despite the big progress in respect to the improved remission rate of a majority of patients (50-60%) under 60 years old, the outcome of Ara-C based treatment is still unsatisfactory as 30-80% of patients relapse eventually after remission [4]. Besides, long-term survival rates continue to be around 30% to 40% for adults, and treatment of patients with relapsed or refractory AML with Ara-C based chemotherapy is unable to produce a prolonged leukemia-free survival in most patients [4,5]. The mechanism behind poor survival of AML patients with Ara-C based treatment still remains unclear.

**Figure 1:**
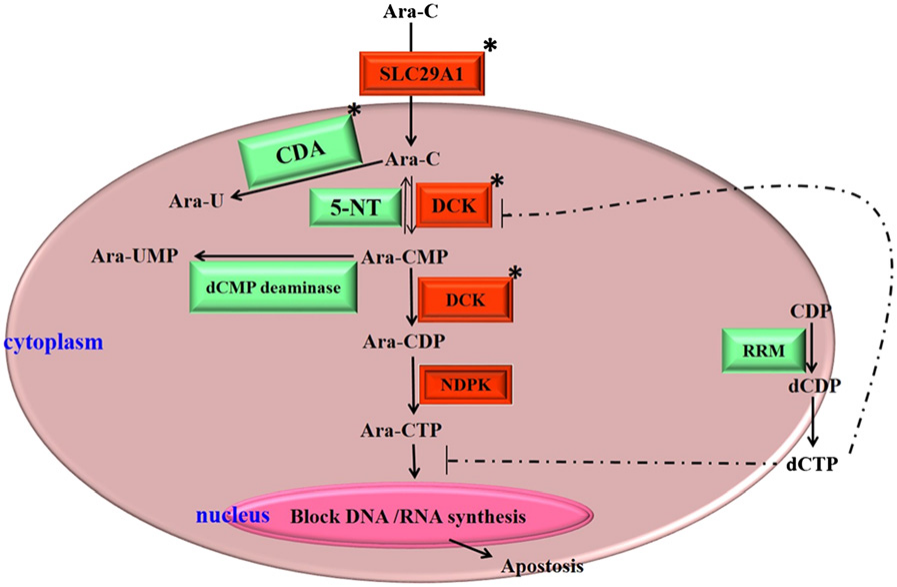
**Schematic description of Ara-C transport and metabolism.** The asterisked letters indicate genes examined in this study.

The cytotoxic effect of Ara-C needs metabolic activation following transport into the cells. When administered in standard doses, Ara-C is transported into cells via membrane transporters including the solute carrier family 29 (nucleoside transporters) member 1 (SLC29A1) [6,7]. High-dose Ara-C diffuses into the cell at a rate higher than that of pump-mediated transport [6,7]. Inside the cell, Ara-C is converted to its active triphosphate form (Ara-CTP) through a series of phosphorylation actions mediated by deoxycytidine kinase (DCK), deoxycytidylate kinase and nucleoside diphosphate kinase (NDPK) [8,9] (Figure 1). DCK is the rate-limiting enzyme in this process. Ara-CTP plays its cytotoxic role by incorporating into DNA to inhibit DNA synthesis in a competitive way, resulting in leukemic cell death [10–13]. Cytoplasmic 5’-nucleotidase (5-NT) dephosphorylates Ara-CMP, a key intermediate, to prevent accumulation of Ara-CTP, which might reduce cellular sensitivity to the cytotoxic activity of Ara-C [14]. CDA can catalyze the hydrolytic deamination of Ara-C to its inactive metabolite 1-B-D-arabinofuranosyluracil (Ara-U). Ribonucleotide reductase (RRM), which consists of 2 subunits, could decrease Ara-C cytotoxycity by catalyzing the de novo synthesis of dNTP which could inhibit the function of DCK [15–18].

Genes involved in Ara-C transport and metabolism, and the potential mechanisms of Ara-C resistance, are investigated in the previous studies. DCK can be inhibited by increased dCTP pool through a negative feedback, and increased CDA function leads to increase in the deamination of Ara-C to AraU [19–21]. Decreased Ara-C transport over the cell membrane into the cytoplasm [6,22] or inactivation of DCK [23,24] can both offset the cytotoxic function of Ara-C. Our previous study showed continuous exposure to Ara-C could induce drug resistance with decreased transcription level of DCK and SLC29A1 as well as elevated mRNA expression of CDA (data not shown). Genetic variations, particularly SNPs, have been identified in these genes involved in Ara-C transport and metabolism [25–27]. Both in vivo and in vitro studies demonstrated that the activities of these enzymes are correlated with polymorphic gene variations [28,29], and some of these SNPs are even highly correlated with treatment response and survival of AML patients with Ara-C based chemotherapy [30–33]. Previous study found that rs329491 of SLC29A1 maybe a favorable survival factor for patients with pancreatic cancer [34]. Another study also showed that rs9394992 may be associated with the survival of patients with non-small cell lung cancer [35]. However, none of these SNPs have been reported in leukemia patients.

To investigate the possible involvement of genes correlating with Ara-C transport and metabolism in patients with chemotherapy-resistant AML, we assessed the pharmacogenomic effects of Ara-C metabolic/transport pathway in AML in this study.

## Results

### Patients’ characteristics and treatment outcomes

Baseline characteristics and treatment results of the 100 AML patients were summarized in Table 1. A total of 51 patients (54.8%) were of normal karyotype. Among the patients who were available for their cytogenetic or molecular information, 14 patients had t (8; 21) (q22; q22). Of the 93 patients detected, 8 patients carried NPM1 mutation, and 5 patients with FLT3 internal tandem duplication (ITD) mutation were identified in 92 patients. The median and mean follow-up duration were 44 and 37.8 months, respectively. 74% (n = 74) of patients achieved remission after one cycle (n = 69) or two cycles (n = 5) of Ara-C based induction chemotherapy. Overall, the five-year DFS and OS rate of the AML patients was (35.7 ± 2.4)%, and (43.2 ± 2.2)%, respectively.

**Table 1:** **Characteristics of AML patients**

**Characteristics**	**∑n**	***n***	***%***	**Median (range)**
Gender (Male/female)	100	42/58	42/58	
Age (years)	100			43 (17–76)
Bone marrow blasts (%)	92			67.3 (37.5-97)
WBC count (x10^9^/L)	95			17.9 (2.5-193.9)
FAB classification	100			
M2		10	10	
M4		48	48	
M5		40	40	
M6		2	2	
Karyotype	93	51/42	54.8/45.2	
Normal/abnormal				
Favorable		20	21.5	
Intermediate		65	69.9	
Adverse		8	8.6	
NPM1	93	8/85	8.6/91.4	
Positive/negative				
FLT-ITD	92	5/87	5.4/94.6	
Positive/negative				

FAB, French-American-British.

### Transcription level of genes involving in Ara-C transport and metabolism

**Figure 2:**
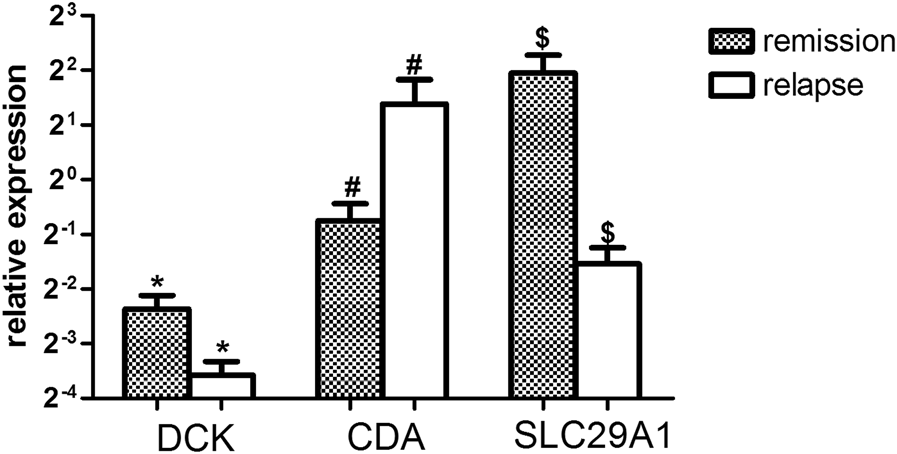
**The mRNA expression of genes related to Ara-C efficacy in AML.** Gene expression levels of DCK, CDA, and SLC29A1 were analyzed by quantitative RT-PCR. β-actin was used as an internal control. *^#^$ indicated statistically significant (P < 0.05).

To detect the transcription of DCK, CDA and SLC29A1, we examined mRNA expression of these genes in the leukemia blasts from the bone marrow of AML patients. The results showed that the RNA expression of DCK and SLC27A1in patients with complete remission was higher than that of non-remission ones, while CDA expression in the remission patients was lower than that of non-remission ones (Figure 2). The similar results was identified in our previous in-vitro study as well (Additional file 1c)

.

### Genotype frequencies of DCK, CDA and SLC29A1 polymorphisms

**Figure 3:**
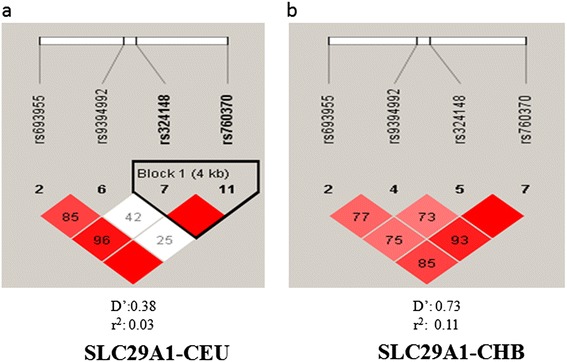
**SLC29A1 Haplotype structure.** The haplotype structure of SLC29A1 was generated based on HapMap Phase II + III Release 27 data. Colors ranging from bright red to light red to white indicate the range of r2 values from high to low. The link of rs9394992 to rs324148 that we identified in the survival analysis for AML patients is in the white **(a)** or light red box **(b)** with r2 < 0.5. CHB: Han Chinese in Beijing, China. CEU: Utah residents with Northern and Western European ancestry from CEPH collection.

19 SNPs (Table 2) of DCK, CDA and SLC29A1 were screened in 100 AML patients and 100 healthy controls, and their genotype frequencies and allele frequencies were summarized (Additional file 2). SNP15 was excluded from analysis since only one genotype was identified in the AML patients and normal healthy controls. Genotype frequencies of the other 18 SNPs were identified in Hardy-Weinberg equilibrium (*χ*^2^ = 0.002-3.590, P = 0.580-0.960). No difference of genotype and allele frequencies of all the 18 SNPs were found between the AML patients and the healthy controls. Genotype frequencies were not significantly different between male and female AML patients. No significant correlation was observed between other AML prognostic factors including WBC at presentation, age or cytogenetic abnormalities, and genotypes of SLC29A1 polymorphic variants. The two SNPs of SLC29A1b, rs324148 and rs9394992, were not in strong linkage disequilibrium (LD) (D’ = 0.73, r^2^ = 0.11) in CHB as well as LD and CEU (D’ = 0.38, r^2^ = 0.03) (Figure 3a, b).

**Table 2:** **Characteristics of 19 SNPs**

**Gene**	**SNP**	**HGVS Names**	**ref SNP ID**	**Global MAF**	**Location**	**Chromosome position**
**DCK**	SNP1	NM_000788.2:c.-201C > T	rs2306744	0.060/130	exon	4:71859352
	SNP2	NM_000788.2:c.207 + 9846A > G	rs12648166	0.458/998	intron	4:71873745
	SNP3	NM_000788.2:c.757-1205C > T	rs4694362	0.467/1015	intron	4:71893864
	SNP4	NM_000788.2:c.165C > T	rs4643786	0.227/494	exon	4:71895260
	SNP5	NM_000788.2:c.207 + 11338A > G	rs7684954	0.197/430	intron	4:71875237
	SNP6	NM_000788.2:c.666-346 T > C	rs936869	0.196/426	intron	4:71892036
	SNP7	NM_000788.2:c.92-1110 T > C	rs3775289	0.193/419	intron	4:71862674
**CDA**	SNP8	NM_000788.2:c.266 + 3264A > G	rs1689924	0.482/1049	intron	1:20934796
	SNP9	NM_000788.2:c.267-4159C > T	rs572529	0.350/761	intron	1:20936176
	SNP10	NM_000788.2:c.267-4087G > A	rs477155	0.279/606	intron	1:20936248
	SNP11	NM_000788.2:c.154 + 1015A > G	rs818202	0.480/1045	intron	1:20916791
	SNP12	NM_000788.2:c.155-7161G > A	rs818199	0.386/840	intron	1:20924260
	SNP13	NM_000788.2:c.266 + 1809G > A	rs10916827	0.345/752	intron	1:20933341
	SNP14	NM_000788.2:c.266 + 2751G > A	rs527912	0.349/759	intron	1:20934283
	SNP15	NM_000788.2:c.208G > A	rs60369023	0.002/4	exon	1:20931474
**SLC29A1**	SNP16	NM_001078174.1:c.30-549 T > C	rs324148	0.228/496	intron	6:44196578
	SNP17	NM_001078174.1:c.1260-201A > G	rs760370	0.344/750	intron	6:44200953
	SNP18	NM_001078174.1:c.29 + 913C > T	rs9394992	0.290/631	intron	6:44195992
	SNP19	NM_001078174.1:c.-54-3077A > G	rs693955	0.189/411	intron	6:44191920

SNP, single-nucleotide polymorphism; rfID., reference SNP identification; HGVS Names, Human Genome Variation Societyname; MAF, minor allele frequency.

### Impact of SNP genotypes on treatment response

**Figure 4:**
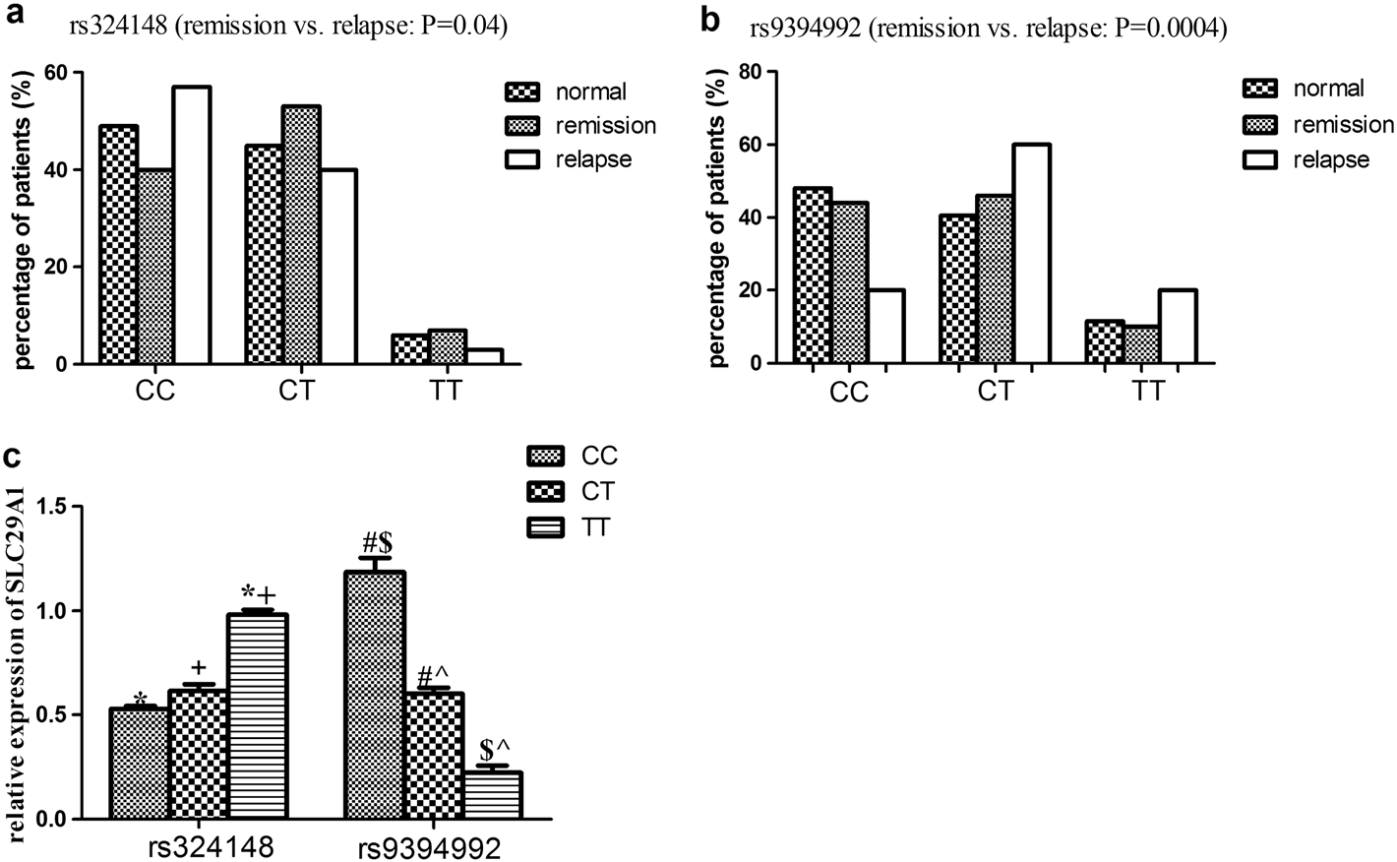
**Genotype frequencies of SNP16 (rs324148) and SNP18 (rs9394992) in AML patients and healthy control, and their impact on the mRNA expression of SLC29A1. a**, genotype frequencies of SNP16 (rs324148) in remission and relapsed patients, and in healthy control. Frequency of genotype CC was higher in relapsed patients than those of CT and TT (P = 0.04); **b**, genotype frequencies of SNP18 (rs9394992) in remission and relapsed patients, and in healthy control. Frequencies of genotype CT and TT were higher in relapsed patients than those of CC (P = 0.0004). No difference of genotype frequencies of both SNPs was observed between healthy control and remission patients. **c**, relative mRNA expression of SLC29A1 in patients with different genotypes of SNP16 and SNP18 by quantitative real time PCR, β-actin was used as an internal control. *# + $^ indicated statistically significant (P < 0.05).

To identify the correlation between genotypes of SNPs with Ara-C based treatment response, all 19 SNPs were screened. The results showed that genotype frequency of genotype CC in SNP16 was higher in relapsed patients, while frequencies of CT and TT were higher in remission ones (P = 0.04). For SNP18, more genotype frequency of genotype CC was found in remission patients, while higher frequencies of genotype CT and TT were found in relapsed ones (P = 0.0004) (Figure 4a, b).

### Impact of genotypes of rs9394992 and rs324148 on SLC29A1 transcription

To further investigate the effect of different genotypes of polymorphic varies on the transcription of SLC29A1, we evaluated the mRNA expression of the rs9394992 and rs324148 of SLC29A1 in AML patients. Higher mRNA expression of genotype TT was observed in rs324148, as compared to that of genotype CC (P < 0.01). Higher expression of genotype CC was observed in rs9394992 compared with genotype CT and TT (P < 0.01) (Figure 4c), which indicated that SNPs might modify Ara-C toxicity through transcription regulation.

### The effect of SNPs on Ara-C based treatment outcomes

**Figure 5:**
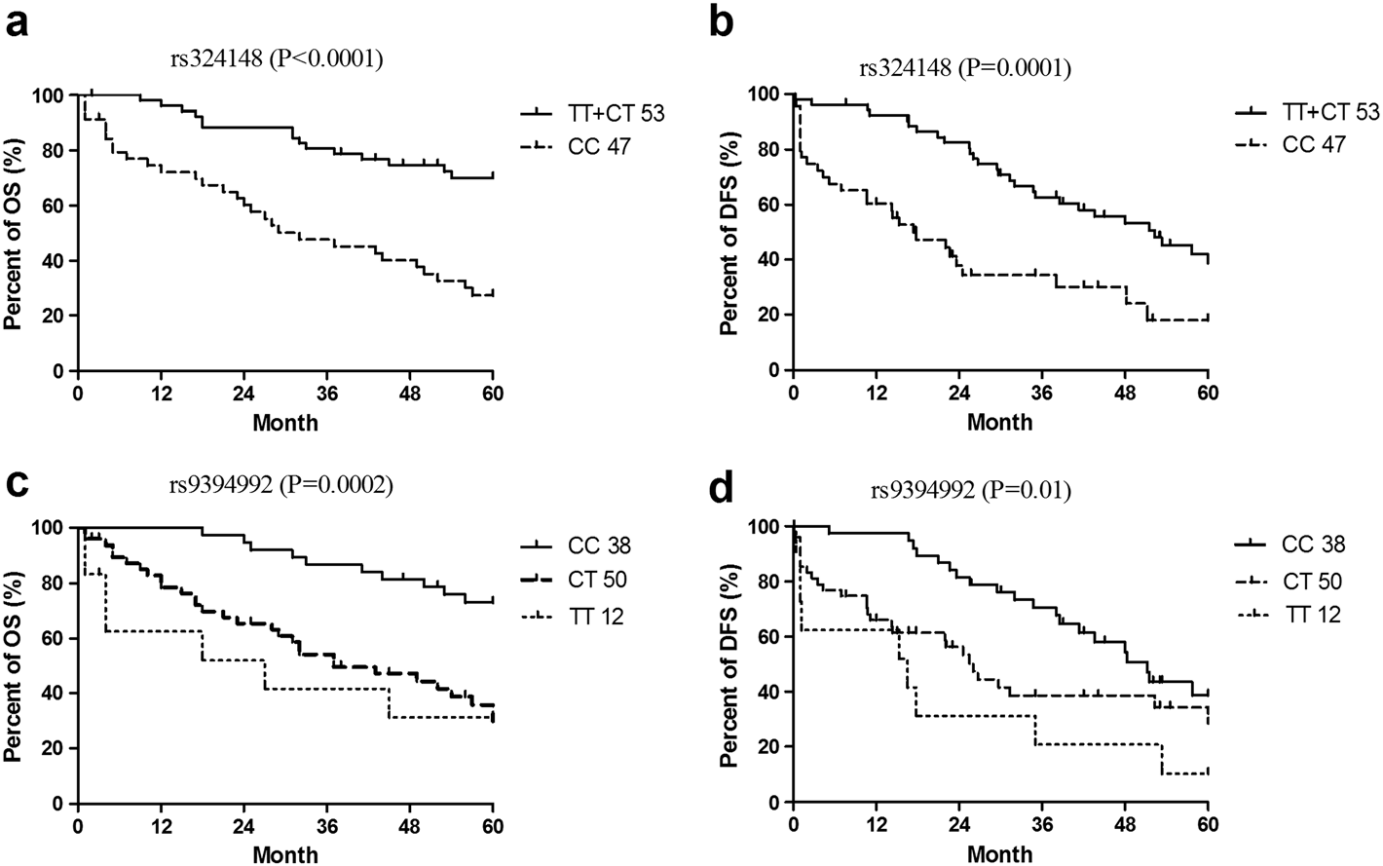
**Univariate analysis of survival rates based on genotypes of SNP16 (rs324148) and SNP19 (rs9394992). a**: effect of SNP16 on OS of AML, **b**: effect of SNP16 on DFS of AML; **c**, effect of SNP18 on OS of AML; **d**, effect of SNP18 on DFS of AML.

**Figure 6:**
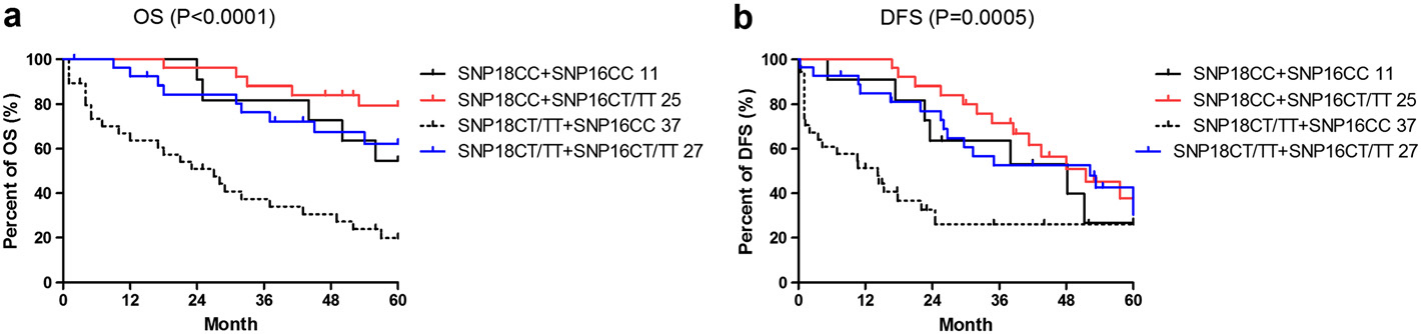
**Effect of SNP-SNP interactions on OS and DFS of patients with AML. a**, combined effects of SNP16 and SNP18 on OS of patients with AML; **b**, combined effects of SNP16 and SNP18 on DFS of patients with AML.

Univariate analysis found that SNP18 (rs9394992) and SNP16 (rs324148) of SLC29A1, were significant prognostic factors to OS and DFS (Figure 5). The CC genotype of SNP16 was significantly associated with shorter overall survival time compared to the CT and TT genotypes (hazard ratio [HR] = 2.997 [95% confidence interval (CI): 1.71-5.27], P = 0.0001) (Figure 5a). The genotype of CC in SNP18 was significantly associated with longer survival time compared to CT (HR = 0.25 [95% CI: 0.075-0.81], P = 0.02) or TT genotypes (HR = 0.43 [95% CI: 0.24-0.78], P = 0.005) (Figure 5c). This association was more significant in patients with both genotype CC of rs324148 and genotype CT + TT of rs9394992 (Figure 6a).

The CC genotype of SNP16 was significantly associated with shorter DFS compared to the CT and TT genotypes (HR = 3.18 [95% CI: 1.76-5.76], P = 0.0001) (Figure 5b). Genotype of CC of SNP18 was associated with longer DFS compared to CT (HR = 0.52 [95% CI: 0.29-0.93], P = 0.03) or TT genotypes (HR = 0.15 [95% CI: 0.05- 0.47], P = 0.001) (Figure 5d). This association was more significant in patients with both genotype CC of rs324148 and genotype CT + TT of rs9394992 (Figure 6b).

### Impact of other variables on Ara-C based treatment outcomes

**Figure 7:**
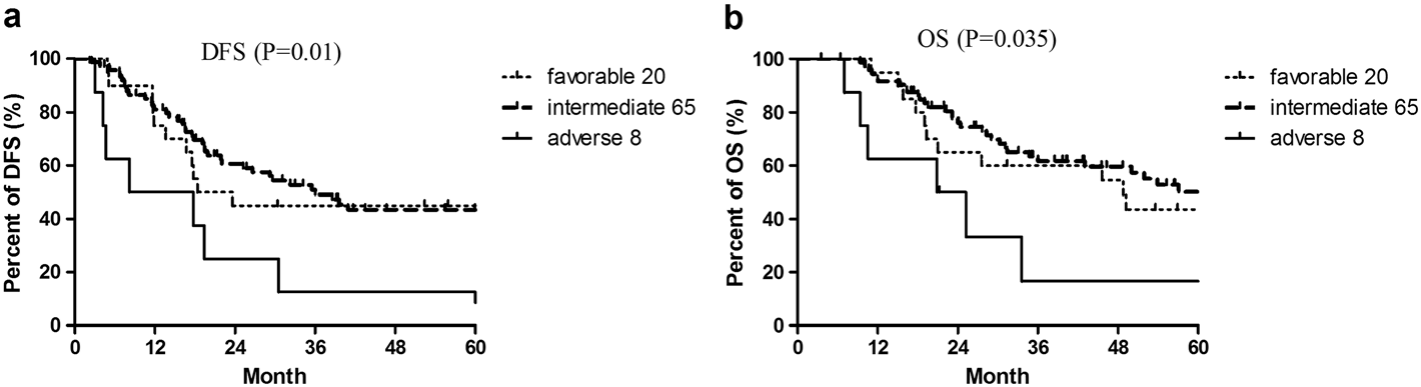
**Univariate analysis of cytogenetic abnormalities on DFS and OS of patients with AML.** Effect of cytogenetic abnormalities on OS and DFS. **a**, Effects of cytogenetic abnormalities on DFS; **b**, Effects of cytogenetic abnormalities on OS. Risk status was evaluated according to the NCCN guidelines version 2.2014 for AML (https://www.nccn.org).

No effects of age, gender and FAB subtypes on the DFS and OS of AML patients were identified (Additional file 3), whereas adverse cytogenetic abnormalities indicated poorer prognosis. However, no difference of DFS and OS was observed between intermediate and favorable cytogenetic abnormalities (Figure 7)

.

## Discussion

It has been demonstrated in the previous studies that cytogenetics and molecular abnormalities were independent predictors for the prognosis of AML patients [36–38]. However, in our study, adverse cytogenetic abnormalities only accounted for 8.6% (n = 93) of patients. The majority of patients was with intermediate and favorable cytogenetics and showed no difference in survival. As Ara-C forms the backbone of the treatment regimen, understanding the contribution of pharmacogenetics to Ara-C response may help dissection of AML with distinct prognosis, so as to individualize chemotherapy and potentially improve the outcomes of AML patients.

In our study, we observed single SNP and SNP-SNP interactions in the Ara-C transport pathway, which could account for the interpatient variability of treatment outcomes in AML patients. When tested independently, both SNP rs324148 and rs9394992 in the SLC29A1gene contributed to Ara-C resistance in patients with AML who received Ara-C based treatment, and they were also significant prognostic factors for survival of these patients. The genotypes of these two SNPs showed various mRNA expressions, which may be responsible for varied response to Ara-C treatment. Although the SNPs in our study located in the intron region of SLC29A1, they affected mRNA expression, which might be due to direct regulation of transcription by altering RNA elongation, splicing or maturation [39–41].

SLC29A1 was expressed in 83% of the AML patients [6]. The results of the studies on the association between SLC29A1 of human AML blasts and clinical drug response were not consistent. Previous data showed that there was no association between SLC29A1 and Ara-C sensitivity [42]. However, some studies demonstrated close correlation of SLC29A1 with Ara-C resistance, treatment response and survival of patients with AML. The inhibition of SLC29A1 expression may induce Ara-C resistance; thereby reduce the overall survival of patients with AML [6,43]. FLT3-ITD indicates poor prognosis in AML, and one of the mechanisms involved was to suppress the expression of SLC29A1 to induce Ara-C resistance in AML patients [44].

Besides SLC29A1, other genes or mechanisms may be responsible for Ara-C resistance. Previous in-vitro studies showed that the expression of DCK was correlated with cellular sensitivity to Ara-C, while decreased DCK activity was observed in Ara-C resistant cell lines [24,45,46]. DCK and 5-NT mRNA expression in leukaemic blasts at diagnosis was correlated with clinical outcome [47], although no alterations in DCK expression and/or activity were observed in resistant and sensitive AML patients [48]. Alternatively spliced forms of DCK with reduced activity were found in Ara-C resistant blasts [48,49], suggesting that DCK may contribute to Ara-C resistance. Our study detected higher DCK expression in leukemia blast in remission patients, but failed to find any difference of polymorphisms between remission and relapsed patients. CDA may be another factor for Ara-C resistance. Elevated CDA activity was correlated with Ara-C resistance [33,50]. CDA could be an independent prognostic parameter for survival in AML patients treated with Ara-C [27]. Other genes, including 5-NT and RRM1 or RRM2, were also found to be involved in Ara-C resistance [14,26,47,51,52].

Our study failed to detect the activity of SLC29A1A in AML patients, in whom we inferred SLC29A1 activity from genotype or mRNA expression correlated with Ara-C sensitivity [43]. In addition, we could not differentiate the difference of survival between different genotype combinations of rs9394992 and rs324148, which might be due to other SNPs. Studies showed DCK rs4694362 (CC genotype) may be a poor prognostic factor for the OS of AML patients. SLC29A1 rs3734703 (AA or AC genotype) together with TYMS rs2612100 (AA genotype) were associated with shorter relapse free survival (RFS) [31]. Polymorphisms within the CDD gene also had an impact on the survival of patients with AML [27]. In AML patients without FLT3-ITD, variant allele of rs10883841 in 5-NT was associated with shorter survival [26]. In addition, polymorphisms of other genes were also identified to be responsible for the prognosis of AML patients [53,54]. Therefore, more parameters need to be included for better prognostic stratification.

Interestingly, our previous study found that fludarabine (Flu) may restore the Ara-C sensitivity in AML blasts (Additional file 1a,b) and increase the cytoxicity of Ara-C (Additional file 1a), partially by increasing the mRNA expression of DCK, SLC29A1, and decreasing mRNA expression of CDA, RRM1 and RRM2 (Additional file 1c). Clinical studies also suggested that Flu may have a beneficial impact on the antileukemic efficacy of Ara-C-based salvage therapy for relapsed and refractory AML [55,56].

In conclusion, cytogenetics might not be enough to predict the prognosis of AML. Polymorphisms in genes related to its Ara-C metabolism may serve as biomarkers for Ara-C sensitivity, treatment response, and prognostic markers in AML, thus individualize chemotherapy and potentially improve outcomes of AML patients.

## Materials and methods

### Patients

From July 2004 to July 2009, 100 Asian Chinese adults with de novo AML other than M3 were randomized to receive low-dose cytarabine (100 mg/m^2^ intravenously for 24 hours, given on day 1–7; n = 100) plus daunorubicin (45 mg/m^2^ intravenously on day 1–3) or indarubicin (10 mg/m^2^ intravenously on day 1–3). High-dose cytarabine (2 g/m^2^ intravenously over 3 hours, given every 12 hours on day 1–3; n = 27) was administered to patients for second cycle induction (n = 4) or for consolidation. All patients were chemonaive at enrollment with leukemia blasts > 70% in the bone marrow. Meanwhile, 100 healthy controls (media age: 46 years, range 14–84 years; n = 50 for male) were also included.

Subjects diagnosed with any other cancers or perniciously administered cytotoxic drugs or radiation was excluded. Primary bone marrow samples were collected after informed consent was obtained from patients or their guardians, in accordance with the Declaration of Helsinki. This study was approved by the institutional research board at Renji Hospital, Shanghai.

### SNP selection and genotyping

Three cytarabine transport and metabolism genes including SLC29A1, DCK and CDA were reported to potentially involve in the response to cytarabine. Based on the database from NCBI (http://www.ncbi.nlm.nih.gov/) and International Hap-Map project (http://hapmap.ncbi.nlm.nih.gov/), 19 candidate SNPs in these genes were initially selected.

Mononuclear cells (MNCs) were purified with Ficoll. Genomic DNA was extracted from MNC samples using standard methods recommended and normalized to 1 μg/μl. Quality and quantity of the extracted DNA was checked on a Nanodrop ND-1000 UV–vis Spectrophotometer (NanoDrop Technologies, Wilmington, DE, USA) by spectral absorption scans from 230 to 350 nm. Genotyping for the all genes’ polymorphism was performed using ligase detection reaction (LDR) assay following the manufacturer’s instructions. Gene-specific polymerase chain reaction (PCR) primers and fluorogenic probes for allelic discrimination were supplied by Shanghai Generay Biotech. PCR cycling and ligation reactions were performed in a GeneAmp PCR System 9700 (Applied Biosystems, Foster City, CA) according to the conditions specified by the manufacturer. Ligation products were analyzed using ABI PRISM® 377 DNA Sequencer (Applied Biosystems, Foster City, CA). Genotyping results were duplicated in 15% of samples with 100% concordance between repeats.

Genomic DNA was extracted from peripheral blood samples of 100 healthy donors using QIAamp blood DNA isolation kits (Qiagen Sciences, Maryland, USA) as per the manufacturer’s protocol. Genotyping was performed as described for the patient population.

### Real time PCR

0.5 × 10^6^ leukemia cells were harvested, and total RNA was extracted using the RNeasy Plus Mini kit (QIAGEN, GmbH, Hilden, Germany) following the manufacturer’s protocol. RNA quality and quantification were assessed using the optical spectrometry 260/280 nm ratio. Subsequently, mRNA was reverse transcribed to cDNA using Applied Biosystems High Capacity Reverse Transcription kit (Applied Biosystems, Foster City, CA). Quantitative RT-PCR was performed for DCK, CDA and SLC, with β-actin as the internal control, using SYBR Premix Ex Taqs (TaKaRa, Kyoto, Japan) on the Roche LightCycler® 480 system (Roche, Mannheim, Germany). Total reaction was carried out in 10 μL volume, which consisted of 5 μL SYBR Premix Ex Taqs Master Mix, 0.1 μL primers (final of 10 nM forward and reverse primers), and 4 μL water, along with 0.8 μL cDNA. The fast thermocycler parameters were: 95°C for 10 seconds, and 40 cycles of 95°C for 5 second then 60°C for 30 seconds and 78°C for 1 second. The qRT-PCR was run in triplicate and individual samples run in triplicate on the RT-PCR plates. Primers were supplied by Sangon Biotech in shanghai.

### Definitions

Disease-free survival (DFS) was defined as time from remission to failure at the end of two courses, relapse or death of any cause. Overall survival (OS) was defined as time from study entry to death of any cause. Complete remission (CR) after induction chemotherapy was defined as: (1) normal values for absolute neutrophil (>1000/mcL) and platelet counts (≥100,000/mcL) independent of transfusion; (2) less than 5% of blast cells, no blasts with Auer rods on bone marrow examination; (3) absence of extramedullary leukemia. Partial remission (PR) was defined as decrease of at least 50% in the percentage of blasts to 5-25% in the bone marrow aspirate and the normalization of blood counts. CR and PR were defined as overall remission. Relapse was defined as marrow infiltration by more than 5% of blast cells in previous normal bone marrow or evidence of extramedullary leukemia. Patients lost to follow-up, survived (for OS) or maintained remission (for DFS) up to the end of the research were censored at their date of last known contact.

### Statistical analysis

Differences of the frequencies between genotypes and alleles in AML (remission and relapsed) patients and normal controls were evaluated using Chi-square test and Fisher’s exact test when data were sparse. The expression of mRNA between different genotypes of SNPs or between different genes was analyzed with *t* test. DFS and OS were calculated using Kaplan-Meier estimates. A cut off P-value of 0.05 was adopted for all statistical analyses. Statistical significance is represented by the two-tailed P values.

## Additional files

Additional file 1:
**Effects of Fludarabine on cytotoxicity of Ara-C and expression of genes involved in Ara-C metabolism and transport in vitro.** a, IC50 of HL60 and Ara-C resistant HL60 cell line (HL60R). IC50 of Ara-C in HL60 was (0.15±0.1)μg/ml, and IC50 of Ara-C in HL60R was (47.87±4.02) μg/ml, P=0.002. b, effects of fludarabine on the cytotoxicity of Ara-C in HL60R cell line. After incubation with fludarabine (20μg/ml) and/or Ara-C (800μg/ml) for 24 hours, cell number was counted using Trypan-blue to exclude dead cells. *^&$#+@ indicate statistically significant (P<0.05).c, relative mRNA expression of DCK, CDA, 5-NT, RRM1, RRM2 and SLC29A1 in HL60 and HL60R cells after 24-hour incubation with Flu and/or Ara-C. mRNA was detected by real time quantitative PCR, and β-actin was used an an internal control. *^&#+@ indicate statistically significant (P<0.05). Additional file 2:
**Genotype/allele frequencies of 19 SNPs of DCK, CDA and SLC29A1 in healthy control and AML patients.** a, b, c showed genotype frequencies of 19 SNPs of DCK, CDA and SLC29A1; d, e, f showed allele frequencies of 19 SNPs of DCK, CDA and SLC29A1. No significant difference of genotype/allele frequencies was observed between the AML patients and healthy controls. Genotype frequencies of the 18 SNPs except SNP15 (only one genotype in AML patients and normal donors) were found to be in Hardy-Weinberg equilibrium (χ^2^ = 0.002-3.590, P =0.580-0.960). Additional file 3:
**Univariate analysis of gender, age, FAB classification on DFS and OS of patients with AML.** a, Effect of age on OS and DFS; b, effect of gender on OS and DFS; c, effect of FAB classification on OS and DFS.
