# Supplementary material for: SLC29A1 single nucleotide polymorphisms as independent prognostic predictors for survival of patients with acute myeloid leukemia: an in vitro study
Source: J Exp Clin Cancer Res. 2014 Nov 15;33(1):90. doi: 10.1186/s13046-014-0090-9 (PMC4234887; doi:10.1186/s13046-014-0090-9)

**Additional file 1: Effects of Fludarabine on cytotoxicity of Ara-C and expression of genes involved in Ara-C metabolism and transport in vitro**. a, IC50 of HL60 and Ara-C resistant HL60 cell line (HL60R). IC50 of Ara-C in HL60 was (0.15±0.1)μg/ml, and IC50 of Ara-C in HL60R was (47.87±4.02) μg/ml, P=0.002. b, effects of fludarabine on the cytotoxicity of Ara-C in HL60R cell line. After incubation with fludarabine (20μg/ml) and/or Ara-C (800μg/ml) for 24 hours, cell number was counted using Trypan-blue to exclude dead cells. *^&$#+@ indicate statistically significant (P<0.05).c, relative mRNA expression of DCK, CDA, 5-NT, RRM1, RRM2 and SLC29A1 in HL60 and HL60R cells after 24-hour incubation with Flu and/or Ara-C. mRNA was detected by real time quantitative PCR, and β-actin was used an an internal control. *^&#+@ indicate statistically significant (P<0.05).


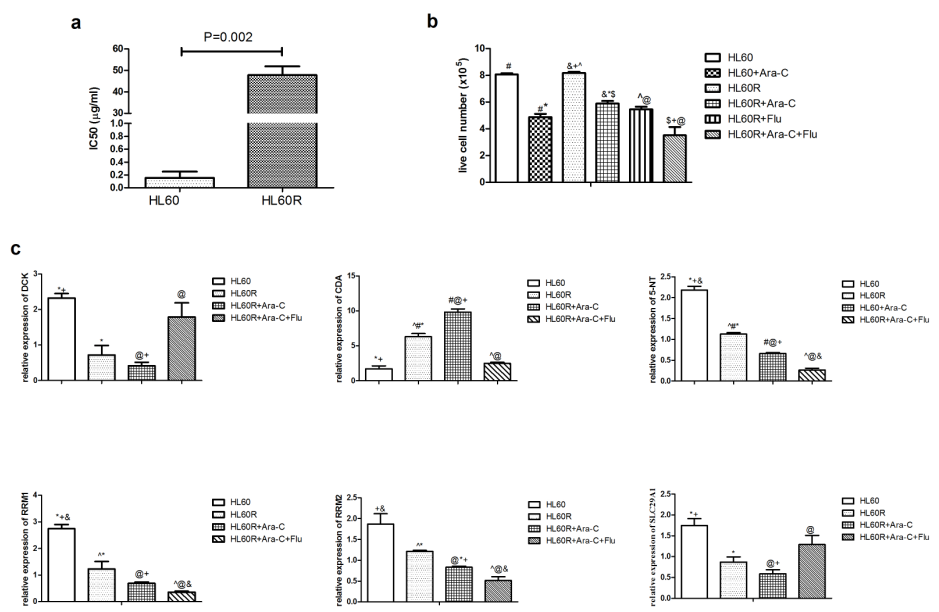

Supplement: Additional file 1: — Effects of Fludarabine on cytotoxicity of Ara-C and expression of genes involved in Ara-C metabolism and transport in vitro. a, IC50 of HL60 and Ara-C resistant HL60 cell line (HL60R). IC50 of Ara-C in HL60 was (0.15±0.1)μg/ml, and IC50 of Ara-C in HL60R was (47.87±4.02) μg/ml, P=0.002. b, effects of fludarabine on the cytotoxicity of Ara-C in HL60R cell line. After incubation with fludarabine (20μg/ml) and/or Ara-C (800μg/ml) for 24 hours, cell number was counted using Trypan-blue to exclude dead cells. *^&$#+@ indicate statistically significant (P<0.05).c, relative mRNA expression of DCK, CDA, 5-NT, RRM1, RRM2 and SLC29A1 in HL60 and HL60R cells after 24-hour incubation with Flu and/or Ara-C. mRNA was detected by real time quantitative PCR, and β-actin was used an an internal control. *^&#+@ indicate statistically significant (P<0.05). [file 13046_2014_90_MOESM1_ESM.doc]
