# Supplementary material for: SLC29A1 single nucleotide polymorphisms as independent prognostic predictors for survival of patients with acute myeloid leukemia: an in vitro study
Source: J Exp Clin Cancer Res. 2014 Nov 15;33(1):90. doi: 10.1186/s13046-014-0090-9 (PMC4234887; doi:10.1186/s13046-014-0090-9)

**Additional file 2:** **Genotype/allele frequencies of 19 SNPs of DCK, CDA and SLC29A1 in healthy control and AML patients**. a, b, c showed genotype frequencies of 19 SNPs of DCK, CDA and SLC29A1; d, e, f showed allele frequencies of 19 SNPs of DCK, CDA and SLC29A1. No significant difference of genotype/allele frequencies was observed between the AML patients and healthy controls. Genotype frequencies of the 18 SNPs except SNP15 (only one genotype in AML patients and normal donors) were found to be in Hardy-Weinberg equilibrium (χ^2^ = 0.002-3.590, P =0.580-0.960).


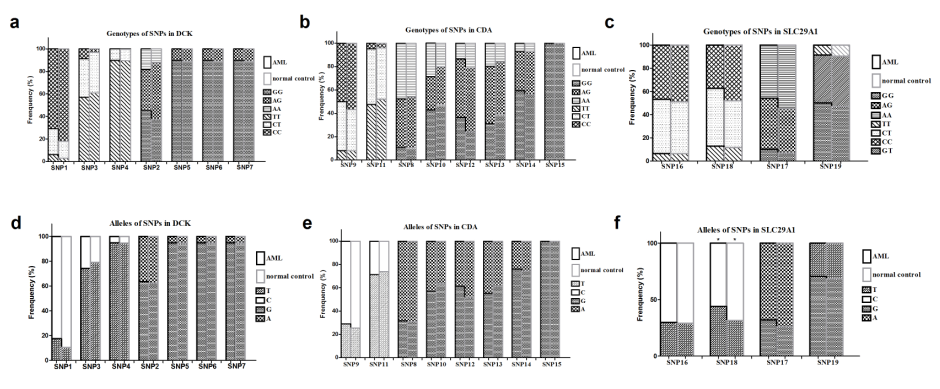

Supplement: Additional file 2: — Genotype/allele frequencies of 19 SNPs of DCK, CDA and SLC29A1 in healthy control and AML patients. a, b, c showed genotype frequencies of 19 SNPs of DCK, CDA and SLC29A1; d, e, f showed allele frequencies of 19 SNPs of DCK, CDA and SLC29A1. No significant difference of genotype/allele frequencies was observed between the AML patients and healthy controls. Genotype frequencies of the 18 SNPs except SNP15 (only one genotype in AML patients and normal donors) were found to be in Hardy-Weinberg equilibrium (χ2 = 0.002-3.590, P =0.580-0.960). [file 13046_2014_90_MOESM2_ESM.doc]
