# Supplementary material for: SLC29A1 single nucleotide polymorphisms as independent prognostic predictors for survival of patients with acute myeloid leukemia: an in vitro study
Source: J Exp Clin Cancer Res. 2014 Nov 15;33(1):90. doi: 10.1186/s13046-014-0090-9 (PMC4234887; doi:10.1186/s13046-014-0090-9)

**Additional file 3: Univariate analysis of gender, age, FAB classification on DFS and OS of patients with AML**. a, Effect of age on OS and DFS; b, effect of gender on OS and DFS; c, effect of FAB classification on OS and DFS.


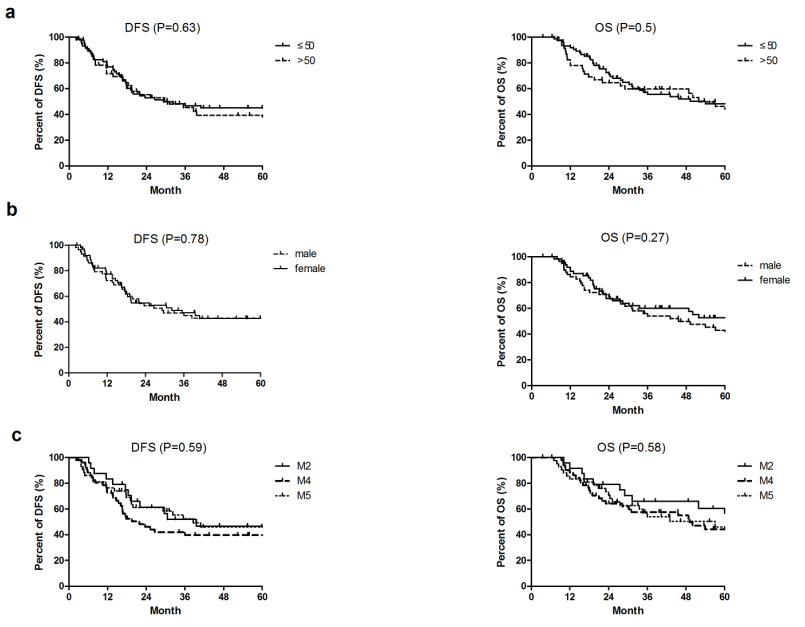

Supplement: Additional file 3: — Univariate analysis of gender, age, FAB classification on DFS and OS of patients with AML. a, Effect of age on OS and DFS; b, effect of gender on OS and DFS; c, effect of FAB classification on OS and DFS. [file 13046_2014_90_MOESM3_ESM.doc]
